# HIV drug resistance amongst children and adolescents with viraemia in Lesotho and Tanzania: a nested analysis in the GIVE MOVE trial

**DOI:** 10.1093/jac/dkag070

**Published:** 2026-03-07

**Authors:** Christof Manuel Schönenberger, Kathrin Haenggi, Isaac Kaumbuthu Ringera, Ezekiel Luoga, Moniek Bresser, Buoang Mothobi, Kuena Mokhele, David Sando, Mamello Molatelle, Lineo Thahane, Dorcas Mnzava, Robert Ndege, Mosa Molapo Hlasoa, Buntshi Paulin Kayembe, Josephine Muhairwe, Tracy Renée Glass, Thomas Klimkait, Maja Weisser, Niklaus Daniel Labhardt, Nadine Tschumi, Jennifer Anne Brown

**Affiliations:** Division of Clinical Epidemiology, Department of Clinical Research, University Hospital Basel, Basel, Switzerland; University of Basel, Basel, Switzerland; Division of Clinical Epidemiology, Department of Clinical Research, University Hospital Basel, Basel, Switzerland; University of Basel, Basel, Switzerland; Division of Clinical Epidemiology, Department of Clinical Research, University Hospital Basel, Basel, Switzerland; University of Basel, Basel, Switzerland; SolidarMed, Partnerships for Health, Maseru, Lesotho; Department of Biomedical Research and Clinical Trials, Ifakara Health Institute, Ifakara, Tanzania; St.Francis Referral Hospital, Ifakara, Tanzania; University of Basel, Basel, Switzerland; Department of Medicine, Swiss Tropical and Public Health Institute, Allschwil, Switzerland; SolidarMed, Partnerships for Health, Maseru, Lesotho; SolidarMed, Partnerships for Health, Maseru, Lesotho; Department of Development Studies, Muhimbili University of Health and Allied Sciences, Dar es Salaam, Tanzania; Seboche Mission Hospital, Seboche, Lesotho; Baylor College of Medicine Children's Foundation Lesotho, Maseru, Lesotho; Baylor College of Medicine, Houston, TX, USA; Department of Biomedical Research and Clinical Trials, Ifakara Health Institute, Ifakara, Tanzania; HPV Laboratory, Department of Gynaecology, Charité—Universitätsmedizin Berlin, Berlin, Germany; University of Basel, Basel, Switzerland; Department of Biomedical Research and Clinical Trials, Ifakara Health Institute, Ifakara, Tanzania; St.Francis Referral Hospital, Ifakara, Tanzania; Department of Medicine, Swiss Tropical and Public Health Institute, Allschwil, Switzerland; Baylor College of Medicine Children's Foundation Lesotho, Maseru, Lesotho; Baylor College of Medicine Children's Foundation Lesotho, Maseru, Lesotho; SolidarMed, Partnerships for Health, Maseru, Lesotho; University of Basel, Basel, Switzerland; Department of Medicine, Swiss Tropical and Public Health Institute, Allschwil, Switzerland; University of Basel, Basel, Switzerland; Molecular Virology, Department of Biomedicine, University of Basel, Basel, Switzerland; University of Basel, Basel, Switzerland; Department of Biomedical Research and Clinical Trials, Ifakara Health Institute, Ifakara, Tanzania; Department of Medicine, Swiss Tropical and Public Health Institute, Allschwil, Switzerland; Division of Infectious Diseases, University Hospital Basel, Basel, Switzerland; Division of Clinical Epidemiology, Department of Clinical Research, University Hospital Basel, Basel, Switzerland; University of Basel, Basel, Switzerland; Division of Clinical Epidemiology, Department of Clinical Research, University Hospital Basel, Basel, Switzerland; University of Basel, Basel, Switzerland; Division of Clinical Epidemiology, Department of Clinical Research, University Hospital Basel, Basel, Switzerland; University of Basel, Basel, Switzerland

## Abstract

**Background:**

Children and adolescents with HIV have lower treatment success than adults. Suboptimal adherence and resistance to antiretroviral therapy (ART) are known aetiological factors. This preplanned analysis in the GIVE MOVE trial (NCT04233242) describes drug resistance patterns in children and adolescents in Lesotho and Tanzania.

**Materials and Methods:**

GIVE MOVE randomized children and adolescents (6 months to below 19 years) with recent viraemia whilst taking ART to genotypic resistance testing (GRT)-informed care or usual care. Here, we conducted additional post-hoc GRT on stored samples from both groups and included participants with at least one successful resistance test. We assessed the number of drugs predicted to be active in participants’ three-drug ART regimens and resistance-associated mutations.

**Results:**

Amongst 137 participants, the majority were female (58%) and lived in Lesotho (77%). At their initial GRT, 69/137 (50%) were receiving protease inhibitor-based, 59/137 (43%) dolutegravir-based and 9/137 (7%) efavirenz-based ART. At that time, 80/137 (58%) participants had three, whilst 8/137 (6%) had two, 36/137 (26%) had one and 13/137 (9%) had no drugs predicted to be active in their regimens. Seventeen (12%) participants had resistance against their ART core agent, including one with high-level dolutegravir resistance.

Across 312 detected resistance-associated mutations (222 major, 90 accessory), 146 conferred resistance to non-nucleoside reverse transcriptase inhibitors, 127 to nucleoside reverse transcriptase inhibitors, 28 to protease inhibitors and 11 to integrase strand transfer inhibitors.

**Conclusion:**

Given that more than half had an ART regimen predicted to be fully active, most viraemia in children and adolescents could not be explained by resistance.

Registration: The GIVE MOVE trial was registered on Clinicaltrials.gov NCT04233242

## Introduction

Globally, 2.4 million children and adolescents aged 0–19 were living with HIV in 2024, 62% of whom lived in Eastern or Southern Africa.^1^ Both treatment coverage and treatment success are lower amongst children and adolescents than amongst adults.^2–4^

Limited treatment options in paediatric formulations, the need for adjustments in dosage due to increasing body weight and numerous practical and social barriers during childhood and adolescence contribute to suboptimal viral suppression in children and adolescents living with HIV.^5,6^ This, alongside vertically transmitted resistance, increases the risk of developing resistance to antiretroviral therapy (ART).^7^ Given the need for lifelong treatment and limited therapeutic options, the emergence of drug resistance is of particular concern amongst children and adolescents. However, in most countries with a high prevalence of HIV, including Lesotho and Tanzania, genotypic resistance testing (GRT) is not routinely available. Therefore, resistance surveillance amongst children and adolescents living with HIV in these countries remains scarce.

The Genotype-Informed Versus Empirical Management of VirEmia (GIVE MOVE) trial investigated whether GRT and expert recommendations to inform management of viraemia would improve clinical and virological treatment outcomes over usual care amongst children and adolescents with HIV in Lesotho and Tanzania.^8,9^ In GIVE MOVE, GRT-informed management did not show a significant benefit over usual care. In this preplanned analysis of GIVE MOVE data, we assess the prevalence of HIV drug resistance in children and adolescents with viraemia.

## Methods

### Study design

GIVE MOVE was an open-label randomized controlled trial conducted at 10 healthcare facilities in Lesotho and Tanzania between March 2020 and July 2023. A total of 284 eligible children and adolescents between 6 months and below 19 years of age with viraemia of at least 400 copies/mL in the last 16 weeks were randomized 1:1 to receive either GRT-informed treatment with expert recommendation (GRT group) or repeat viral load testing after enhanced adherence counselling and empirical onward treatment (usual care group). Participants in the intervention group also received enhanced adherence counselling. Amongst 144 participants in the GRT group, 84 received a full or partial GRT at the time of enrolment, 58 had resuppressed to below 400 copies/mL by enrolment (impeding GRT, but indicating susceptibility to ART) and for two participants GRT failed despite a viral load above 400 copies/mL). After 36 weeks, outcomes in the GRT group did not differ significantly from usual care for the composite primary endpoint, which included death, hospitalization, new WHO HIV stage 4 events or the absence of documented viral suppression of less than 50 copies/mL.^8,9^

In this preplanned analysis, we performed additional GRT on stored GIVE MOVE samples from both groups and assessed drug resistance levels and resistance-associated mutations (RAMs).

### Setting

In routine care, before the rollout of dolutegravir-based ART, children above 3 years of age and adolescents were initiated on ART containing efavirenz as a core agent. Younger children were initiated on ritonavir-boosted lopinavir-based ART, which was also used as second-line treatment after virologic treatment failure.^10^ Since 2018, the World Health Organization (WHO) has recommended the use of dolutegravir-based ART as first-line therapy for adults, adolescents and children.^11^

In Lesotho and Tanzania, dolutegravir rollout began in 2019 for people newly initiating ART or transitioning from nevirapine- or efavirenz-based ART.^12,13^ The main transition from ritonavir-boosted lopinavir-based ART followed in 2022–2023.^13,14^

### Ethics

GIVE MOVE was approved in Lesotho by the National Health Research Ethics Committee (first approval identifier 229-2019), and in Tanzania by the National Institute for Medical Research (NIMR/HQ/R.8a/Vol IX/3442), the Tanzania Medicines and Medical Devices Authority (TMDA0020/CTR/0003/03) and the Ifakara Health Institute Institutional Review Board (12-2020). All participants or their caregivers consented to this study. For this secondary analysis, no separate ethical approval was needed.

GIVE MOVE was registered with ClinicalTrials.gov (NCT04233242). This analysis is reported following the STROBE recommendations.^15^

### Participants and measurements

The GIVE MOVE trial included children and adolescents in Lesotho and Tanzania in care with HIV viraemia of at least 400 copies/mL in the previous 16 weeks; in this analysis, we included GIVE MOVE participants with at least one successful GRT. GRT consisted of Sanger sequencing of the protease, reverse transcriptase and, in individuals taking integrase strand transfer inhibitors (INSTIs)-based ART, the integrase region of *pol* as per the respective laboratories’ routine protocols. If the *pol* regions relevant to the ART regimen at the time of sampling were sequenced successfully, GRT was considered successful. For this analysis, we included GRT results performed at enrolment in the GRT group (trial intervention), post-hoc GRT results from stored blood samples taken at enrolment in the usual care group and GRT results from viremic samples taken at or after 36 weeks from both groups. In the following, we refer to the first available GRT result per participant as the *initial* GRT. If a participant has a second GRT result or a subsequent viral load measurement, this is referred to as the *follow-up* GRT or *follow-up* viral load, respectively.

All data used for this analysis were collected within the GIVE MOVE trial using the electronic data capture software MACRO version 4.8.1 (Ennov, Paris, France). We used the Stanford HIV drug resistance database (version 9.6) to classify resistance levels (high-level resistance, intermediate resistance, low-level resistance, potential low-level resistance and susceptible) to the antiretroviral drugs taken at the time of sampling.^16^ We excluded non-RAMs in RAM positions and classified all RAMs as *accessory* or *major* following the Stanford HIV drug resistance database. We considered a drug to be fully active if GRT predicted susceptibility or potential low-level resistance. Further, we assessed immune deficiency scores based on CD4 cell count and percentages with age-dependent ranges (Appendix Table 1, available as Supplementary data at *JAC* Online).

### Statistical analysis

We summarized participant characteristics descriptively. We used medians/interquartile ranges (IQR) for continuous variables and frequencies/percentages for categorical variables. For data management and analysis, we used R, version 4.4.1 (2024-06-14). All packages used are listed (Appendix Table 2).

## Results

### Study population

**Figure 1.**
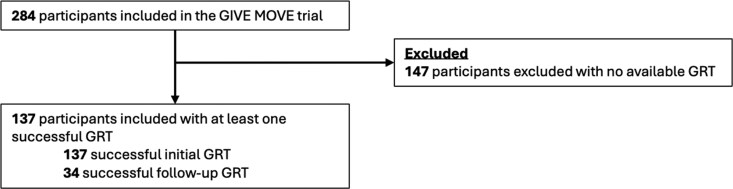
GRT, genotypic resistance test.

Amongst 284 GIVE MOVE participants, 137 participants had at least one successful GRT and were included in this analysis (Figure 1). Of these, 34 participants had a *follow-up* GRT result over the course of the trial.

Table 1 provides information on participants’ characteristics. The median age was 13 years (IQR 5–16). Over half of the participants were female (79/137; 57.7%) and the majority lived in Lesotho (105/137; 76.6%). At the *initial* GRT, 39/137 (28.5%) participants self-reported having missed ≥1 dose of ART in the past 4 weeks, 33/137 (24.1%) had advanced or severe immune deficiency and 98/137 (71.5%) had a viral load of 1000 copies/mL or more. Most participants were taking a protease inhibitor (PI)-based regimen (69/137; 50.4%) or a dolutegravir-based regimen (59/137; 43.1%) and nine (6.6%) were taking an efavirenz-based regimen (Table 1). At the time of their *initial* GRT, the median time on ART was 5 (IQR 3–10) years and the median time on their current regimen was 2 (IQR 1–3) years.

**Table 1.** Participants’ characteristics at time of initial GRT

Characteristic demographics	Overall (*n* = 137)
Age (in years)	
Median [IQR]	13 [5, 16]
12 years and above	78 (56.9%)
Below 12 years	59 (43.1%)
Sex	
Female	79 (57.7%)
Male	58 (42.3%)
Country	
Lesotho	105 (76.6%)
Tanzania	32 (23.4%)
Trial group	
Usual care group	57 (41.6%)
GRT group	80 (58.4%)

Clinical and treatment data	Overall (*n* = 137)
Missed ≥1 dose of ART over the last 4 weeks (self-/caregiver-report)	
No	79 (57.7%)
Yes	39 (28.5%)
Unknown	19 (13.9%)
Period of ≥2 days without ART intake over the past 4 weeks (self-/caregiver-report)	
No	85 (62.0%)
Yes	33 (24.1%)
Unknown	19 (13.9%)
Current WHO stage	
T1	134 (97.8%)
T2	2 (1.5%)
T3	0 (0%)
T4	1 (0.7%)
CD4 count at ART initiation (in cells/mm^3^)	
Median [IQR]	601 [356.5, 1130.8]
<200	6 (4.4%)
200–499	15 (10.9%)
≥500	33 (24.1%)
Missing	83 (60.6%)
WHO stage at ART initiation	
T1	81 (59.1%)
T2	18 (13.1%)
T3	16 (11.7%)
T4	12 (8.8%)
Unknown	10 (7.3%)
CD4 count at enrollment to the GIVE MOVE trial (in cells/mm^3^)	
Median [IQR]	582 [381, 851.5]
<200	8 (5.8%)
200–499	31 (22.6%)
≥500	60 (43.8%)
Missing	38 (27.7%)
Immune deficiency score at enrollment to the GIVE MOVE trial	
Not significant	51 (37.2%)
Mild	26 (19.0%)
Advanced	19 (13.9%)
Severe	14 (10.2%)
Missing	27 (19.7%)
Viral load (in copies/mL)	
Median [min, max]	8390 [1380, 38223.5]
<400	11 (8.0%)
400–999	14 (10.2%)
1000–99 999	81 (59.1%)
≥100 000	17 (12.4%)
Missing	14 (10.2%)
ART regimen type	
PI-based	69 (50.4%)
ABC/3TC/LPVr	55 (40.1%)
AZT/3TC/LPVr	9 (6.6%)
TDF/3TC/LPVr	3 (2.2%)
ABC/3TC/ATVr	2 (1.5%)
INSTI-based	59 (43.1%)
ABC/3TC/DTG	31 (22.6%)
TDF/3TC/DTG	27 (19.7%)
AZT/3TC/DTG	1 (0.7%)
NNRTI-based	9 (6.6%)
ABC/3TC/EFV	5 (3.6%)
AZT/3TC/EFV	4 (2.9%)
Time on ART (in years)	
Median [IQR]	5 [3, 10]
Time on current ART (in years)	
Median [IQR]	2 [1, 3]
PI-based regimen	3 [2, 4]
INSTI-based regimen	2 [1, 2]
NNRTI-based regimen	4 [2, 8]

3TC, lamivudine; ABC, abacavir; ART, antiretroviral therapy; ATVr, ritonavir-boosted atazanavir; AZT, zidovudine; DTG, dolutegravir; EFV, efavirenz; INSTI, integrase strand transfer inhibitor; IQR, interquartile range; LPVr, ritonavir-boosted lopinavir; NA, not available; NNRTI, nonnucleoside reverse transcriptase inhibitor; NRTI, nucleoside reverse transcriptase inhibitor; PI, protease inhibitor; TDF, tenofovir disoproxil fumarate; WHO, World Health Organization.

### Resistance levels at initial GRT

At the *initial* GRT, 80/137 (58.4%) participants were predicted to have a fully active ART regimen, 8/137 (5.8%) were predicted to have two active drugs, 36/137 (26.3%) were predicted to have one active drug and 13/137 (9.5%) were predicted to have no active drugs in their regimen (Table 2). Resistance against the core agent was detected in 17/137 (12.4%) participants: eight taking efavirenz-, six ritonavir-boosted lopinavir -, one ritonavir-boosted atazanavir- and one dolutegravir-based ART. Appendix Table 3 summarizes all participants with resistance against the ART core agent. GRT predicted resistance to lamivudine in 56/137 (40.9%) and to the second nucleoside reverse transcriptase inhibitor (NRTI) in 46/137 (33.6%) participants.

**Table 2.** Resistance outcomes at the initial GRT

Resistance data	Overall (*n* = 137)
Number of drugs predicted to be active	
0	13 (9.5%)
1	36 (26.3%)
2	8 (5.8%)
3	80 (58.4%)
Predicted lamivudine resistance level	
High-level resistance	56 (40.9%)
Susceptible	81 (59.1%)
Predicted second NRTI resistance level	
High-level resistance	20 (14.6%)
Intermediate resistance	2 (1.5%)
Low-level resistance	24 (17.5%)
Susceptible	91 (66.4%)
Predicted core agent resistance level	
High-level resistance^a^	16 (11.7%)
Intermediate resistance^b^	1 (0.7%)
Susceptible	120 (87.6%)

Mutation data	Overall (*n* = 137)
Number of people with any resistance-associated mutation	88 (64.2%)
Atleast one major resistance-associated mutation	83 (60.6%)
At least one accessory but no major resistance-associated mutation	5 (3.6%)
Number of people with an NRTI mutation	60 (43.8%)
Number of people with an NNRTI mutation	72 (52.6%)
Number of people with a PI mutation	11 (8.0%)
Number of people with an INSTI mutation	7 (5.1%)

ATVr, ritonavir-boosted atazanavir; DTG, dolutegravir; EFV, efavirenz; INSTI, integrase strand transfer inhibitor; LPVr, ritonavir-boosted lopinavir; NNRTI, nonnucleoside reverse transcriptase inhibitor; NRTI, nucleoside reverse transcriptase inhibitor; PI, protease inhibitor.

^a^EFV (*n* = 9), LPVr (*n* = 5), DTG (*n* = 1), ATVr (*n* = 1).

^b^LPVr (*n* = 1).

### Resistance-associated mutations at the initial GRT

Across the 137 participants’ *initial* GRTs, 321 RAMs were detected, including 228 major and 93 accessory mutations. Of these mutations, 150, 129, 29 and 13 were associated with resistance against non-nucleoside reverse transcriptase inhibitors (NNRTIs), NRTIs, PIs and INSTIs, respectively (Appendix Figure 1 and Appendix Table 4).

**Figure 2.**
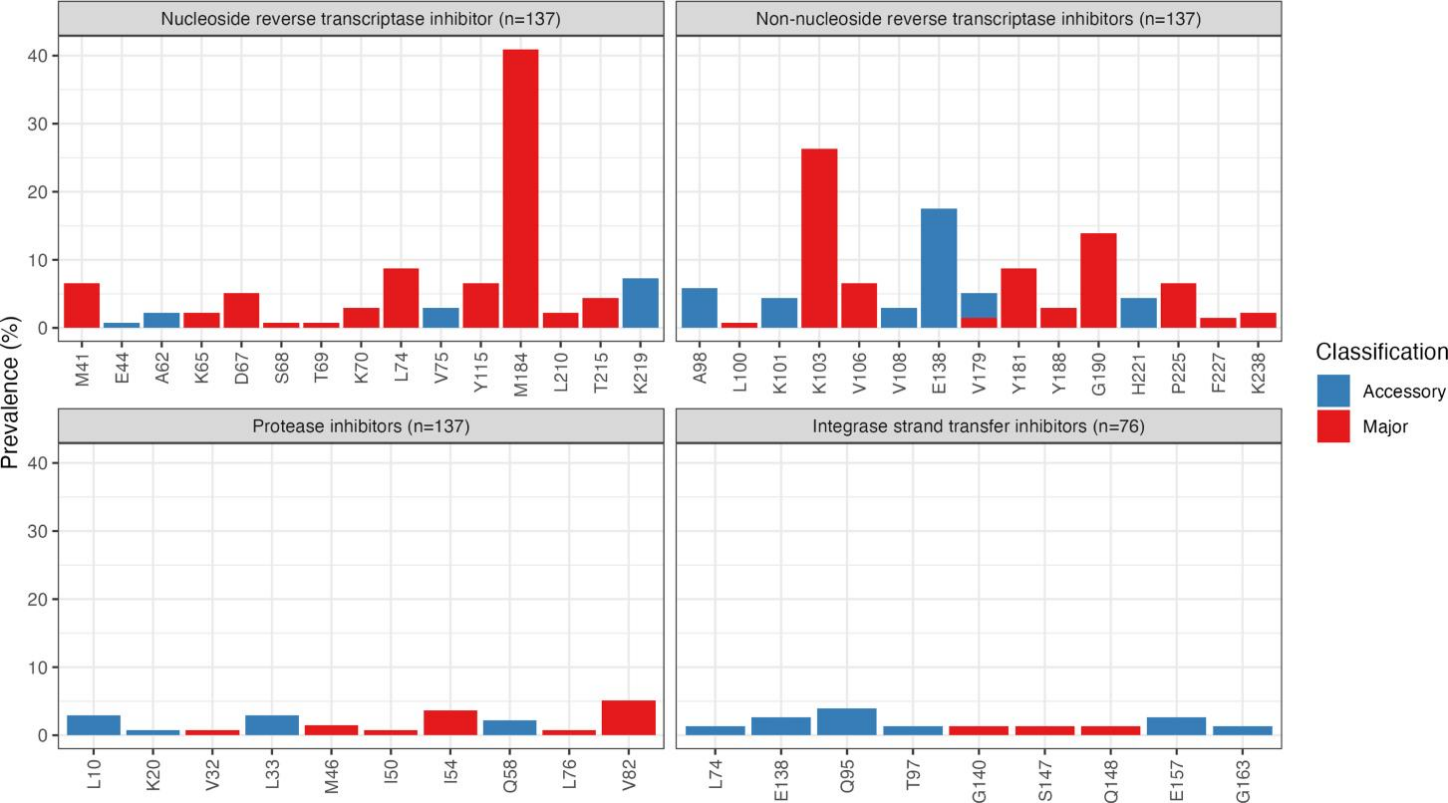
Resistance-associated mutation positions stratified by mutation classification (accessory or major) at the *initial* GRT. The denominator is the total number of successful *initial* GRTs per drug class. The numerator is the number of detected resistance-associated mutations in the respective position.

Figure 2 and Table 2 summarize the prevalence of detected major and accessory RAMs by codon position. At the time of *initial* GRT, 88/137 (64.2%) participants had at least one RAM, 83/137 (60.6%) had at least one major RAM, and 60/137 (43.8%), 72/137 (52.6%), 11/137 (8.0%) and 7/137 (5.1%) had at least one RAM against NRTIs, NNRTIs, PIs, or INSTIs respectively. M184V, conferring high-level resistance to lamivudine, was the most frequently detected mutation associated with NRTI resistance, detected in 56/137 (40.9%) participants (Appendix Figure 1). The most frequently detected mutation associated with NNRTI resistance was K103N, detected in 33 out of 137 participants (24.1%). Major RAMs to PIs (M46I, *n* = 2; I54 V, *n* = 5; V82A, *n* = 7; L76 V, *n* = 1; I50L, *n* = 1; V32L, *n* = 1) and INSTIs (G140A, *n* = 1; S147G, *n* = 1; Q148K, *n* = 1) were less common.

### Longitudinal development of resistance over the study period

**Figure 3.**
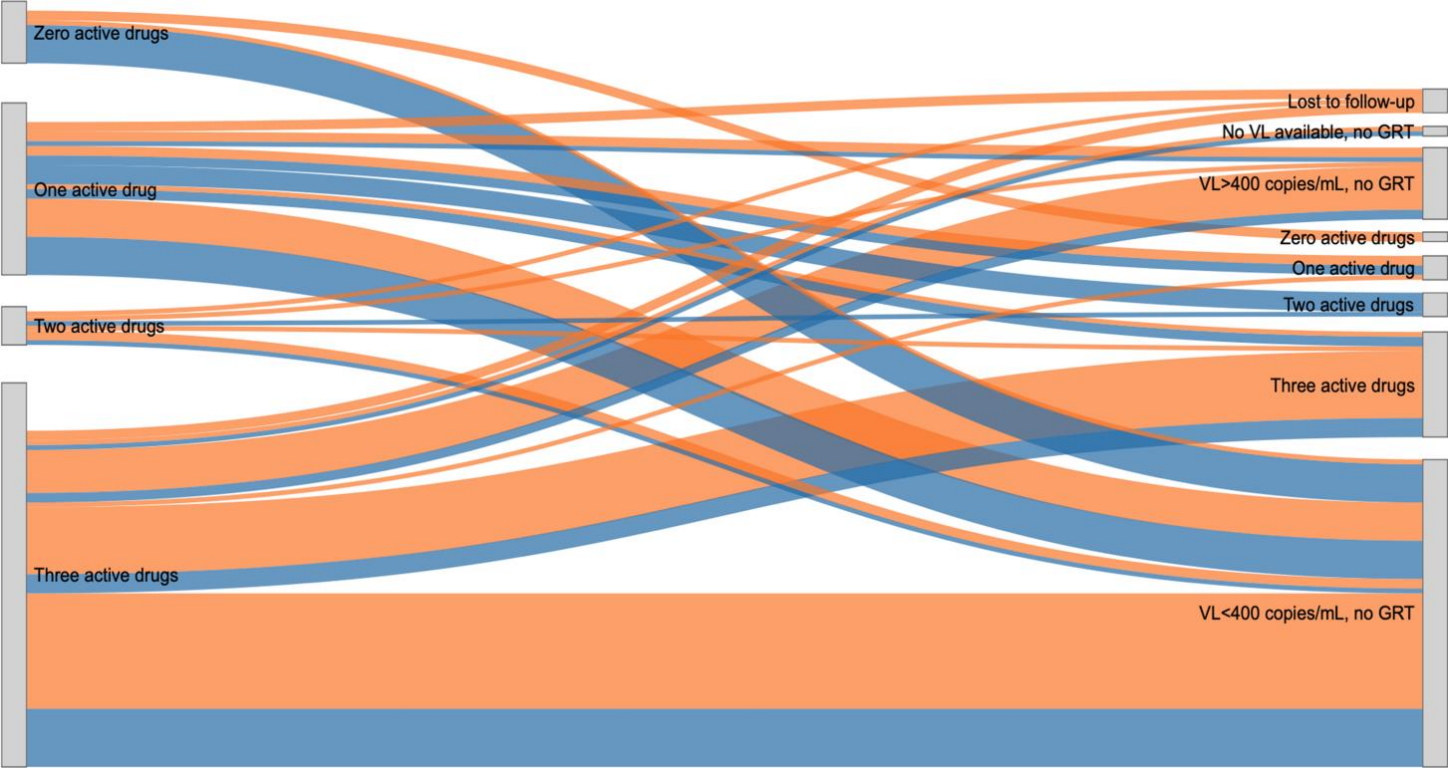
On the left side, at the *initial* GRT, there are 137 participants with a successful GRT defined as a GRT including all sequences of their current ART regimen. The right side shows viral load or GRT outcomes at the *follow-up* time point. Five participants were lost to follow-up or transferred out by the end of the study. Seventeen participants had no *follow-up* information since their first successful GRT was at the end of the GIVE MOVE study (no flow is shown for these participants). A total of 16 participants had a treatment change involving the ART backbone only, 14 participants had a treatment change involving the ART core agent only and 17 participants had a treatment change involving the ART backbone and core agent. Legend: A blue flow indicates a change to the ART regimen and orange indicates no change to the ART regimen.

Figure 3 provides an overview of the longitudinal development of resistance, stratified by treatment change. *Follow-up* VL or GRT information was available for 113 of the 137 participants; for 17, no *follow-up* information was available as the *initial* GRT was completed at the end of the trial, five participants were lost to follow-up or transferred out by the end of the trial and two participants remained in care but had no viral load at trial completion.

66/113 (48.2%) participants with subsequent VL or GRT data had three active drugs at the *initial* GRT. Of these, 18/66 (27.3%) received a regimen change during the study. At *follow-up*, 36/66 (54.5%) had resuppressed to <400 copies/mL, 18/66 (27.3%) maintained three active drugs, 1/66 (1.5%) acquired treatment-relevant resistance (in the form of a new M184V mutation, conferring high-level resistance to lamivudine and low-level resistance to abacavir in the ART backbone) and 11/66 (16.7%) had a *follow-up* viral load >400 copies/mL without a successful *follow-up* GRT.

Amongst the 47/113 (41.6%) participants with fewer than three active drugs at the *initial* GRT who had subsequent VL or GRT data, 27/47 (57.4%) received a regimen change. 28/47 (59.6%) resuppressed to <400 copies/mL (17/28 and 11/28 with and without treatment change, respectively), including one participant with zero active drugs at the *initial* GRT who resuppressed without ART modification. The remaining 19/47 (40.4%) had viraemia ≥400 copies/mL at *follow-up*. Of these, 11 had a successful GRT which predicted fewer than three active drugs, four had no successful GRT and four had no detectible resistances (two with and two without treatment change).

Appendix Table 5 provides an overview on RAMs at both time points in participants with two available GRTs.

## Discussion

We assessed antiretroviral drug resistance patterns amongst children and adolescents with HIV viraemia in the GIVE MOVE trial who received GRT as part of the trial intervention or post-hoc using stored samples. Amongst the 137 children and adolescents included in this analysis, 62.0% had at least one RAM and 41.6% had predicted resistance against at least one drug of their current ART regimen, including 12.4% with resistance to their ART core agent.

The prevalence of RAMs in GIVE MOVE was slightly lower than in a 2024 systematic review encompassing 162 datasets from 52 countries, which reported a RAM prevalence of 74.2% amongst treatment-experienced children and adolescents.^17^ In this review, the prevalence of RAMs by ART class was 65.2% for NRTI RAMs, 56.4% for NNRTI RAMs, 17.1% for PI RAMs and 2.8% for INSTI RAMs. In GIVE MOVE, these numbers were 43.8%, 52.6%, 8.0% and 5.1%, respectively. The Opt4Kids trial, which tested an intervention package including GRT upon detection of viraemia amongst children and adolescents in Kenya, also reported a slightly higher RAM prevalence than GIVE MOVE: of 107 GRTs conducted for 81 individuals, all samples harboured at least one RAM, with 57.0%, 82.2% and 8.4% harbouring at least one mutation associated with NRTI, NNRTI and PI resistance, respectively (INSTI resistance was not tested).^18^ Finally, the ODYSSEY trial compared dolutegravir-based paediatric first- and second-line ART with the respective standard of care at the time, mostly containing efavirenz as the core agent in first- and ritonavir-boosted lopinavir in second-line ART.^19^ Amongst 58 participants who developed virological failure by Week 96 whilst taking first-line ART, 50.0% had predicted resistance against NRTIs, 75.9% against NNRTIs, 3.4% against PI and none against INSTIs. Amongst 75 participants with virological failure by Week 96 whilst taking second-line ART, 77.3% had predicted resistance against NRTIs, 88.0% against NNRTIs, 6.7% against PIs and 6.7% against INSTIs.

GIVE MOVE aimed to inform the optimal management of HIV viraemia in children and adolescents, finding no difference between GRT-informed management and usual care. It was conducted contemporaneously with the rollout of dolutegravir-based ART. Since the dolutegravir rollout, the optimal management of viraemia and potential resistance has been uncertain. This is reflected in heterogeneous guidelines across African countries.^20^ Some countries, including Tanzania, align with WHO guidelines recommending a regimen switch without GRT upon confirmed virological failure with dolutegravir,^20^ though actual implementation differs from these guidelines. Given the low prevalence of dolutegravir resistance observed in this and other studies,^21–24^ with this approach most switches—often to a more complex regimen—would be unnecessary. Other countries, including Lesotho, recommend continuing dolutegravir-based ART and performing GRT only after ≥2 years, since resistance is considered unlikely within that timeframe.^25^ However, recent evidence contradicts this assumption.^21^ Furthermore, in Lesotho, GRT remains *de facto* unavailable.^8^ In the present study, whilst PI and dolutegravir resistance were rare, eight individuals taking PI- or dolutegravir-based ART who had resistance to their core agent would have been unlikely to receive a regimen change in usual care in the absence of GRT. Finally, several African countries recommend GRT upon sustained viraemia without limitations regarding ART duration,^20^ which may not reflect actual technical and financial capacity.

Modelling studies have compared these three approaches, concluding that routine use of GRT would mitigate the emergence of dolutegravir resistance,^26^ improve clinical outcomes,^27,28^ and be cost-effective.^27,28^ To date, this is not supported by randomized trials including GIVE MOVE.^9,29^ This uncertainty around managing viraemia on dolutegravir has two key implications. First, more targeted approaches that consider prognostic factors for resistance could increase the potential benefit of GRT. Second, better tools to measure and support adherence are needed. In the present study, 57.7% self-reported never having missed an ART dose in the last 4 weeks (with a further 13.9% unknown), whereas GRT showed that only 12.4% had intermediate- or high-level resistance to their core agent. For the others, suboptimal adherence remains the likely cause of viraemia, highlighting the limitations of self-reported measures of adherence. One potential avenue is the use of point-of-care urine drug level testing, which could enable earlier detection and management of non-adherence, and triage costly GRT to individuals with viraemia and detectable ART.^30–33^ Finally, with dolutegravir resistance predicted to rise, continued surveillance will remain essential.

Our study has limitations. First, as GRT is not part of routine care in Lesotho and Tanzania, the GIVE MOVE trial obtained informed consent for and enrolled children and adolescents with recent viraemia of whom some had resuppressed by enrolment, prohibiting GRT. The sample size for the present analysis was further limited by incomplete sample storage and limited access to testing of the integrase region of *pol*. Second, we do not have access to pre-treatment data and thus cannot always distinguish between transmitted and acquired resistance. Third, the study was conducted contemporaneously with the dolutegravir rollout. Thus, at the time of the initial GRT, only 59 individuals in this study were taking dolutegravir (for a median of 2 years), which has since largely replaced the other ART regimens included in this study. Fourth, whilst we previously reported several sociodemographic factors,^8^ we did not collect data on factors such as caregiver dependence, regimen fatigue, stigma or formulation-related barriers, which limited our ability to further explore determinants of adherence in the study population.

In conclusion, most children and adolescents with HIV viraemia included in this analysis harboured at least one RAM and treatment-relevant resistance was prevalent at 41.6%. However, only 12.4% had resistance to the core agent, and most viraemia amongst children and adolescents could not be explained by drug resistance. This highlights the importance of improving adherence support for this age group, as well as the need for more targeted approaches to implement costly resistance testing.

## Supplementary Material

dkag070_Supplementary_Data

## Supplementary data

Figure S1 Appendix and Appendices Table S1 to Table S5 are available as Supplementary data at *JAC* Online.
