# Supplementary material for: HIV drug resistance amongst children and adolescents with viraemia in Lesotho and Tanzania: a nested analysis in the GIVE MOVE trial
Source: J Antimicrob Chemother. 2026 Mar 7;81(4):dkag070. doi: 10.1093/jac/dkag070 (PMC13016912; doi:10.1093/jac/dkag070)
Supplement: dkag070_Supplementary_Data [file dkag070_supplementary_data.docx]

**Appendix**

**HIV drug resistance among children and adolescents with viremia in Lesotho and Tanzania: A secondary analysis of the GIVE MOVE trial**

Christof Manuel Schönenberger, Kathrin Haenggi, Isaac Kaumbuthu Ringera, Ezekiel Luoga, Moniek Bresser, Buoang Mothobi, Kuena Mokhele, David Sando, Mamello Molatelle, Lineo Thahane, Dorcas Mnzava, Robert Ndege, Mosa Molapo Hlasoa, Buntshi Paulin Kayembe, Josephine Muhairwe, Tracy Renée Glass, Thomas Klimkait, Maja Weisser, Niklaus Daniel Labhardt, Nadine Tschumi, and Jennifer Anne Brown

# Appendix table 1: Age-adjusted immune deficiency score by CD4 cell count or CD4 percentage.

| **Classification** | **<1 year** | **≥1 year, <3 years** | **≥3 years, <5 years** | **≥5 years, <10 years** | **≥10 years** |
| --- | --- | --- | --- | --- | --- |
| **Not significant** | ≥35% | ≥30% | ≥25% | ≥500 cells/ mm^3^ | ≥500 cells/ mm^3^ |
| **Mild** | ≥30%, <35% | ≥25%, <30% | ≥20%, <25% | ≥350 cells/ mm^3^, <500 cells/ mm^3^ | ≥350 cells/ mm^3^, <500 cells/ mm^3^ |
| **Advanced** | ≥25%, <30% | ≥20%, <25% | ≥15%, <20% | ≥200 cells/ mm^3^, <350 cells/ mm^3^ | ≥200 cells/ mm^3^, <350 cells/ mm^3^ |
| **Severe** | <25% or <1500 cells/ mm^3^ | <20% or <750 cells/ mm^3^ | <15% or <350 cells/ mm^3^ | <15% or <200 cells/ mm^3^ | <200 cells/ mm^3^ |

Adapted from World Health Organization case definitions of HIV for surveillance and revised clinical staging and immunological classification of HIV-related disease in adults and children 2007. CD4: cluster of differentiation 4.

Appendix table 2: References to R packages

| Name | Reference |
| --- | --- |
| tidyverse | Wickham H, Averick M, Bryan J, Chang W, McGowan LD, François R, Grolemund G, Hayes A, Henry L, Hester J, Kuhn M, Pedersen TL, Miller E, Bache SM, Müller K, Ooms J, Robinson D, Seidel DP, Spinu V, Takahashi K, Vaughan D, Wilke C, Woo K, Yutani H (2019). “Welcome to the tidyverse.” _Journal of Open Source Software_,*4*(43), 1686. doi:10.21105/joss.01686 <https://doi.org/10.21105/joss.01686>. |
| writexl | Ooms J (2024). _writexl: Export Data Frames to Excel 'xlsx' Format_. R package version 1.5.0, <https://CRAN.R-project.org/package=writexl>. |
| readxl | Wickham H, Bryan J (2023). _readxl: Read Excel Files_. R package version 1.4.3, <https://CRAN.R-project.org/package=readxl>. |
| kableExtra | Zhu H (2024). _kableExtra: Construct Complex Table with 'kable' and Pipe Syntax_. R package version 1.4.0, <https://CRAN.R project.org/package=kableExtra>. |
| tableone | Yoshida K, Bartel A (2022). _tableone: Create 'Table 1' to Describe Baseline Characteristics with or without Propensity Score Weights_. R package version  0.13.2, <https://CRAN.R-project.org/package=tableone>. |
| lubridate | Garrett Grolemund, Hadley Wickham (2011). Dates and Times Made Easy with lubridate. Journal of Statistical Software, 40(3), 1-25. URL https://www.jstatsoft.org/v40/i03/. |
| officer | Gohel D, Moog S (2024). _officer: Manipulation of Microsoft Word and PowerPoint Documents_. R package version 0.6.6, <https://CRAN.R-project.org/package=officer>. |
| table1 | Rich B (2023). _table1: Tables of Descriptive Statistics in HTML_. R package version 1.4.3, <https://CRAN.R-project.org/package=table1>. |
| networkD3 | Allaire J, Gandrud C, Russell K, Yetman C (2017). _networkD3: D3 JavaScript Network Graphs from R_. R package version 0.4, <https://CRAN.R-project.org/package=networkD3>. |
| htmlwidgets | Vaidyanathan R, Xie Y, Allaire J, Cheng J, Sievert C, Russell K (2023). _htmlwidgets: HTML Widgets for R_. R package version 1.6.4, <https://CRAN.R-project.org/package=htmlwidgets>. |
| flextable | Gohel D, Skintzos P (2024). _flextable: Functions for Tabular Reporting_. R package version 0.9.6, <https://CRAN.R-project.org/package=flextable>. |
| gt | Iannone R, Cheng J, Schloerke B, Hughes E, Lauer A, Seo J, Brevoort, K, Roy O (2025). _gt: Easily Create Presentation-Ready Display <https://CRAN.R-project.org/package=gt>. |

Appendix table 3: Characteristics of participants with resistance against the ART core agent.

3TC, lamivudine; ABC, abacavir; ART, antiretroviral therapy; ATVr, ritonavir-boosted atazanavir; AZT, zidovudine; DRVr, ritonavir-boosted darunavir; DTG, dolutegravir; EFV, efavirenz; GRT, genotypic resistance test; INSTI, integrase strand transfer inhibitor; LPVr, ritonavir-boosted lopinavir; NNRTI, non-nucleoside reverse transcriptase inhibitor; NRTI, nucleoside reverse transcriptase inhibitor; PI, protease inhibitor; RAL, raltegravir; TDF, tenofovir disoproxil fumarate

| **ID** | **Clinical and demographic information at initial GRT** | **ART regimen** | **Viral load** | **Resistance levels (ART regimen underlined)** | **Resistance-associated mutations** |
| --- | --- | --- | --- | --- | --- |
| 1 | Female,  16 years old  Years since HIV diagnosis: 10  Years on ART: 8  Years on current ART: 3 | **At initial GRT:**  TDF/3TC/DTG  **At follow-up:**  Missing^1^ | **At initial GRT:**  400-999 copies/mL  **At follow-up:**  Missing^1^ | **At initial GRT:**  LPVr: Susceptible  DRVr: Susceptible  ATVr: Susceptible  EFV: High-level resistance  ABC: Intermediate resistance  AZT: High-level resistance  3TC: High-level resistance  TDF: High-level resistance  DTG: High-level resistance  RAL: High-level resistance  **At follow-up GRT:**  Missing^1^ | **At initial GRT:**  PI: None  NRTI: D67N, K70R, M184V, K219Q  NNRTI: A98G, K103N, V108I, K238T  INSTI: E138K, G140A, S147G, Q148K  **At follow-up GRT:**  Missing^1^ |
| 2 | Female,  16 years old  Years since HIV diagnosis: 1  Years on ART: 1  Years on current ART: 1 | **At initial GRT:**  ABC/3TC/EFV  **At follow-up:**  TDF/3TC/DTG | **At initial GRT:**  1’000-99’9999 copies/mL  **At follow-up:**  <50 copies/ml | **At initial GRT:**  LPVr: Susceptible  DRVr: Susceptible  ATVr: Susceptible  EFV: High-level resistance  ABC: High-level resistance  AZT: Susceptible  3TC: High-level resistance  TDF: Susceptible  DTG: missing  RAL: missing  **At follow-up GRT:**  Not available (resuppressed) | **At initial GRT:**  PI: None  NNRTI: V106M, E138A, V179E, G190A, H221Y  NRTI: L74V, Y115F, M184V  INSTI: missing  **At follow-up GRT:**  Not available (resuppressed) |
| 3 | Male,  12 years old  Years since HIV diagnosis: 2  Years on ART: 2  Years on current ART: 2 | **At initial GRT:**  ABC/3TC/EFV  **At follow-up:**  DTG/LPVr | **At initial GRT:**  50-400 copies/mL  **At follow-up:**  <50 copies/ml | **At initial GRT:**  LPVr: Susceptible  DRVr: Susceptible  ATVr: Susceptible  EFV: High-level resistance  ABC: Intermediate resistance  AZT: Intermediate resistance  3TC: High-level resistance  TDF: Susceptible  DTG: missing  RAL: missing  **At follow-up GRT:**  Not available (resuppressed) | **At initial GRT:**  PI: None  NNRTI: G190E, G190Q  NRTI: M41L, D67N, V75T, M184V, K219E  INSTI: missing  **At follow-up GRT:**  Not available (resuppressed) |
| 4 | Male,  9 years old  Years since HIV diagnosis: 3  Years on ART: 3  Years on current ART: 3 | **At initial GRT:**  ABC/3TC/EFV  **At follow-up:**  AZT/3TC/DTG | **At initial GRT:**  1’000-99’9999 copies/mL  **At follow-up:**  <50 copies/ml | **At initial GRT:**  LPVr: Susceptible  DRVr: Susceptible  ATVr: Susceptible  EFV: High-level resistance  ABC: High-level resistance  AZT: Susceptible  3TC: High-level resistance  TDF: Susceptible  DTG: Susceptible  RAL: Susceptible  **At follow-up GRT:**  Not available (resuppressed) | **At initial GRT:**  PI: None  NNRTI: K101H, V106M, G190A  NRTI: L74V, Y115F, M184V  INSTI: None  **At follow-up GRT:**  Not available (resuppressed) |
| 5 | Female,  7 years old  Years since HIV diagnosis: 4  Years on ART: 4  Years on current ART: 2 | **At initial GRT:**  ABC/3TC/LPVr  **At follow-up:**  ABC/3TC/DTG | **At initial GRT:**  ≥99’999 copies/mL  **At follow-up:**  50-400 copies/ml | **At initial GRT:**  LPVr: High-level resistance  DRVr: Susceptible  ATVr: High-level resistance  EFV: Susceptible  ABC: Low-level resistance  AZT: Susceptible  3TC: High-level resistance  TDF: Susceptible  DTG: missing  RAL: missing  **At follow-up GRT:**  Not available (resuppressed) | **At initial GRT:**  PI: M46I, I54V, V82A, Q58E  NNRTI: None  NRTI: M184V, K219Q  INSTI: missing  **At follow-up GRT:**  Not available (resuppressed) |
| 6 | Female,  18 years old  Years since HIV diagnosis: 9  Years on ART: 8  Years on current ART: 8 | **At initial GRT:**  AZT/3TC/EFV  **At follow-up:**  TDF/3TC/DTG | **At initial GRT:**  50-400 copies/mL  **At follow-up:**  <50 copies/ml | **At initial GRT:**  LPVr: Susceptible  DRVr: Susceptible  ATVr: Susceptible  EFV: High-level resistance  ABC: Low-level resistance  AZT: Susceptible  3TC: High-level resistance  TDF: Susceptible  DTG: missing  RAL: missing  **At follow-up GRT:**  Not available (resuppressed) | **At initial GRT:**  PI: None  NNRTI: K103N, V106M, E138A  NRTI: M184V  INSTI: missing  **At follow-up GRT:**  Not available (resuppressed) |
| 7 | Male,  19 years old  Years since HIV diagnosis: 12  Years on ART: 10  Years on current ART: 10 | **At initial GRT:**  AZT/3TC/EFV  **At follow-up:**  TDF/3TC/DTG | **At initial GRT:**  Missing  **At follow-up:**  1’000-99’9999 copies/ml | **At initial GRT:**  LPVr: Susceptible  DRVr: Susceptible  ATVr: Susceptible  EFV: High-level resistance  ABC: Low-level resistance  AZT: Susceptible  3TC: High-level resistance  TDF: Susceptible  DTG: Susceptible  RAL: Susceptible  **At follow-up GRT:**  Missing | **At initial GRT:**  PI: None  NNRTI: V106I, Y188L  NRTI: M184V  INSTI: None  **At follow-up GRT:**  Missing |
| 8 | Male,  2 years old  Years since HIV diagnosis: 2  Years on ART: 2  Years on current ART: 2 | **At initial GRT:**  ABC/3TC/LPVr  **At follow-up:**  Missing^1^ | **At initial GRT:**  1’000-99’9999 copies/ml  **At follow-up:**  Missing^1^ | **At initial GRT:**  LPVr: High-level resistance  DRVr: Susceptible  ATVr: Intermediate resistance  EFV: High-level resistance  ABC: High-level resistance  AZT: Susceptible  3TC: High-level resistance  TDF: Susceptible  DTG: missing  **At follow-up GRT:**  Missing^1^ | **At initial GRT:**  PI: L10F, I54V, Q58E, V82A  NNRTI: K103N, P225H  NRTI: L74V, Y115F, M184V  INSTI: missing  **At follow-up GRT:**  Missing^1^ |
| 9 | Male,  4 years old  Years since HIV diagnosis: 4  Years on ART: 4  Years on current ART: 4 | **At initial GRT:**  ABC/3TC/LPVr  **At follow-up:**  ABC/3TC/LPVr | **At initial GRT:**  1’000-99’9999 copies/mL  **At follow-up:**  <50 copies/ml | **At initial GRT:**  LPVr: Intermediate resistance DRVr: Susceptible  ATVr: Low-level resistance  EFV: High-level resistance  ABC: High-level resistance  AZT: Susceptible  3TC: High-level resistance  TDF: Intermediate resistance  DTG: Susceptible  RAL: Susceptible  **At follow-up GRT:**  Not available (resuppressed) | **At initial GRT:**  PI: V82A  NNRTI: K103N  NRTI: K65R, M184V  INSTI: None  **At follow-up GRT:**  Not available (resuppressed) |
| 10 | Male,  18 years old  Years since HIV diagnosis: 4  Years on ART: 4  Years on current ART: 4 | **At initial GRT:**  AZT/3TC/EFV  **At follow-up:**  Missing^1^ | **At initial GRT:**  1’000-99’999 copies/ml  **At follow-up:**  Missing^1^ | **At initial GRT:**  LPVr: Susceptible  DRVr: Susceptible  ATVr: Susceptible  EFV: High-level resistance  ABC: Low-level resistance  AZT: Susceptible  3TC: High-level resistance  TDF: Susceptible  DTG: missing  RAL: missing  **At follow-up GRT:**  Missing^1^ | **At initial GRT:**  PI: None  NNRTI: V106M, V179D, F227L  NRTI: V75I, M184V  INSTI: missing  **At follow-up GRT:**  Missing^1^ |
| 11 | Female,  5 years old  Years since HIV diagnosis: 4  Years on ART: 4  Years on current ART: 4 | **At initial GRT:**  ABC/3TC/LPVr  **At follow-up:**  AZT/3TC/RAL | **At initial GRT:**  1’000-99’999 copies/mL  **At follow-up:**  <50 copies/ml | **At initial GRT:**  LPVr: High-level resistance DRVr: Susceptible  ATVr: High-level resistance  EFV: High-level resistance  ABC: High-level resistance  AZT: Susceptible  3TC: High-level resistance  TDF: Susceptible  DTG: missing  RAL: missing  **At follow-up GRT:**  Not available (resuppressed) | **At initial GRT:**  PI: M46I, I54V, V82A, L10F, K20T  NNRTI: Y188L  NRTI: L74V, Y115F, M184V  INSTI: missing  **At follow-up GRT:**  Not available (resuppressed) |
| 12 | Female,  12 years old  Years since HIV diagnosis: 2  Years on ART: 2  Years on current ART: 2 | **At initial GRT:**  ABC/3TC/EFV  **At follow-up:**  AZT/3TC/DTG | **At initial GRT:**  ≥99’999 copies/mL  **At follow-up:**  <50 copies/ml | **At initial GRT:**  LPVr: Susceptible  DRVr: Susceptible  ATVr: Susceptible  EFV: High-level resistance  ABC: High-level resistance  AZT: Susceptible  3TC: High-level resistance  TDF: Potential low-level resistance  DTG: missing  RAL: missing  **At follow-up GRT:**  Not available (resuppressed) | **At initial GRT:**  PI: None  NNRTI: K101E, Y181C, G190S  NRTI: L74V, Y115F, M184V, K219E  INSTI: missing  **At follow-up GRT:**  Not available (resuppressed) |
| 13 | Female,  17 years old  Years since HIV diagnosis: 8  Years on ART: 8  Years on current ART: 8 | **At initial GRT:**  ABC/3TC/EFV  **At follow-up:**  TDF/3TC/DTG | **At initial GRT:**  1’000-99’999 copies/mL  **At follow-up:**  <50 copies/ml | **At initial GRT:**  LPVr: Susceptible  DRVr: Susceptible  ATVr: Susceptible  EFV: High-level resistance  ABC: High-level resistance  AZT: Susceptible  3TC: High-level resistance  TDF: Susceptible  DTG: missing  RAL: missing  **At follow-up GRT:**  Not available (resuppressed) | **At initial GRT:**  PI: None  NNRTI: K103N, V106M  NRTI: L74V, Y115F, M184V  INSIT: missing  **At follow-up GRT:**  Not available (resuppressed) |
| 14 | Male,  10 years old  Years since HIV diagnosis: 10  Years on ART: 10  Years on current ART: 10 | **At initial GRT:**  AZT/3TC/LPVr  **At follow-up:**  AZT/3TC/LPVr | **At initial GRT:**  400-999 copies/mL  **At follow-up:**  1’000-99’9999 copies/ml | **At initial GRT:**  LPVr: High-level resistance  DRVr: Intermediate resistance  ATVr: Intermediate resistance  EFV: Susceptible  ABC: Intermediate resistance  AZT: High-level resistance  3TC: High-level resistance  TDF: Low-level resistance  DTG: Susceptible  RAL: Susceptible  **At follow-up GRT:**  LPVr: High-level resistance  DRVr: Intermediate resistance  ATVr: High-level resistance  EFV: Susceptible  ABC: Intermediate resistance  AZT: Intermediate resistance  3TC: High-level resistance  TDF: Susceptible  DTG: missing  RAL: missing | **At initial GRT:**  PI: L10F, L33F, I54V, Q58E, L76V, V82A  NNRTI: None  NRTI: M41L, M184V, T215F  INSTI: None  **At follow-up GRT:**  PI: L10F,L33F,M46I,I54V,Q58E,L76V,V82A  NNRTI: None  NRTI: M41L, D67N, K70R, M184V  INSTI: missing |
| 15 | Male,  17 years old  Years since HIV diagnosis: 14  Years on ART: 14  Years on current ART: 13 | **At initial GRT:**  AZT/3TC/EFV  **At follow-up:**  Missing^2^ | **At initial GRT:**  1’000-99’999 copies/mL  **At follow-up:**  Missing^2^ | **At initial GRT:**  LPVr: Susceptible  DRVr: Susceptible  ATVr: Susceptible  EFV: High-level resistance  ABC: Susceptible  AZT: Susceptible  3TC: Susceptible  TDF: Susceptible  DTG: missing  RAL: missing  **At follow-up GRT:**  Missing^2^ | **At initial GRT:**  PI: None  NNRTI: K103N, E138G, K238T  NRTI: None  INSTI: missing  **At follow-up GRT:**  Missing ^2^ |
| 16 | Male,  7 years old  Years since HIV diagnosis: 7  Years on ART: 7  Years on current ART: 6 | **At initial GRT:**  ABC/3TC/LPVr  **At follow-up:**  ABC/3TC/LPVr | **At initial GRT:**  1’000-99’9999 copies/mL  **At follow-up:**  1’000-99’999 copies/ml | **At initial GRT:**  LPVr: High-level resistance  DRVr: Susceptible  ATVr: Intermediate resistance  EFV: Intermediate resistance  ABC: Low-level resistance  AZT: Low-level resistance  3TC: High-level resistance  TDF: Susceptible  DTG: Susceptible  RAL: Low-level resistance  **At follow-up GRT:**  LPVr: Intermediate resistance DRVr: Susceptible  ATVr: Intermediate resistance  EFV: Intermediate resistance ABC: Low-level resistance  AZT: Low-level resistance  3TC: High-level resistance  TDF: Susceptible  DTG: missing  RAL: missing | **At initial GRT:**  PI: L10F, I54V, V82A  NNRTI: A98G, E138A, Y181C  NRTI: D67G, M184V, K219E  INSTI: E157Q, G163K  **At follow-up GRT:**  PI: I54V, V82A  NNRTI: A98G, E138A, Y181C  NRTI: D67G, M184V, K219E  INSTI: missing |
| 17 | Female,  16 years old  Years since HIV diagnosis: 16  Years on ART: 15  Years on current ART: 2 | **At initial GRT:**  ABC/3TC/ATVr  **At follow-up:**  TDF/3TC/DTG | **At initial GRT:**  1’000-99’999 copies/mL  **At follow-up:**  <50 copies/ml | **At initial GRT:**  LPVr: Low-level resistance  DRVr: Susceptible  ATVr: High-level resistance  EFV: High-level resistance  ABC: High-level resistance  AZT: High-level resistance  3TC: High-level resistance  TDF: Low-level resistance  DTG: missing  RAL: missing  **At follow-up GRT:**  Not available (resuppressed) | **At initial GRT:**  PI: I50L, V82A  NNRTI: A98G,V179T,Y181C,G190A  NRTI: D67N, L74I, M184V, T215F, K219E  INSTI: missing  **At follow-up GRT:**  Not available (resuppressed) |

^1^No follow up available since the first successful GRT was at the end of the GIVE MOVE study.

^2^Lost to follow-up during the study period.

Appendix figure 1: Resistance-associated mutations stratified by mutation classification (accessory or major) at the initial GRT**.**

The denominator is the total number of successful *initial* GRTs per drug class. The numerator is the number of the respective resistance-associated mutations detected.

**
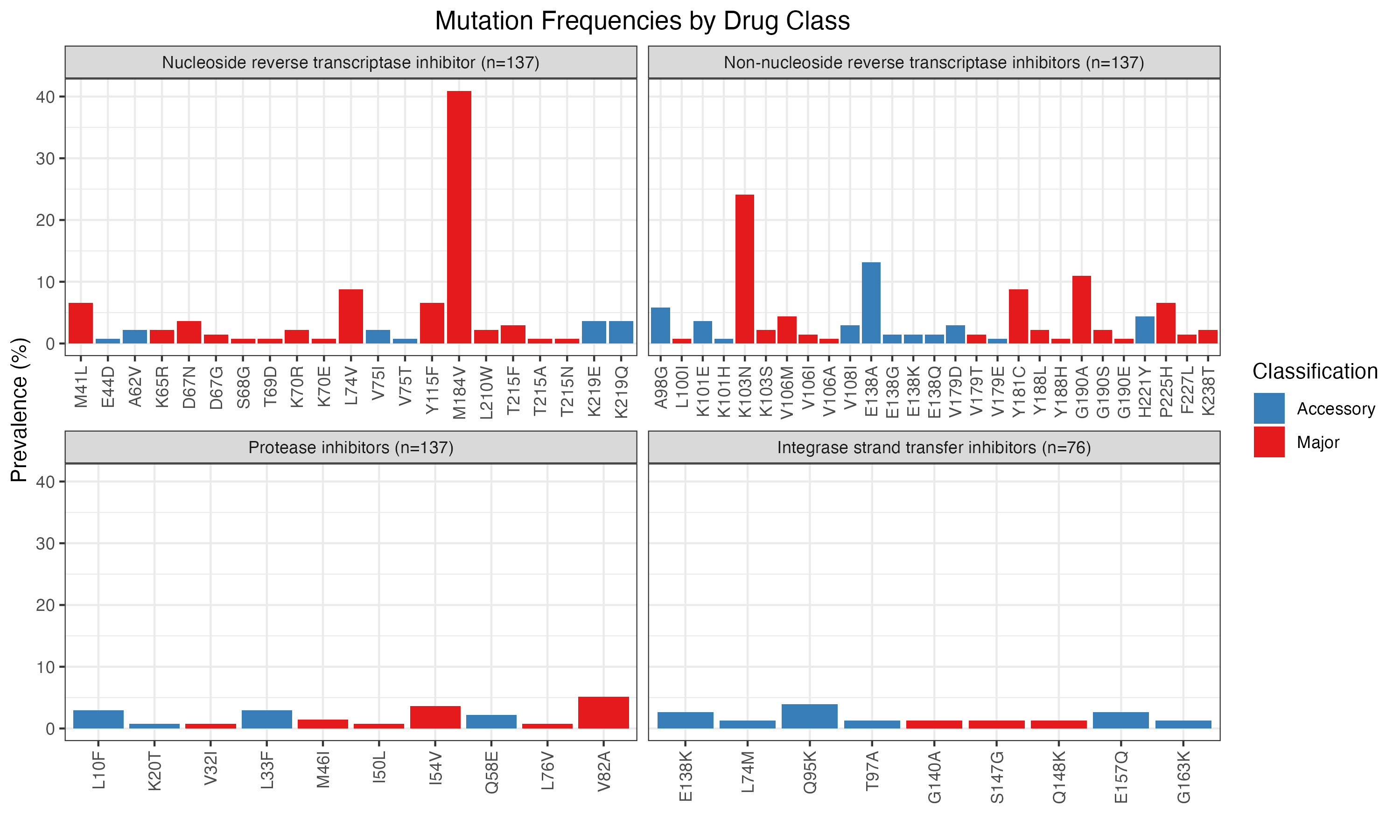
**

Appendix table 4: Detected resistance-associated mutations at the initial GRT (n=321). GRT, genotypic resistance test; INSTI, integrase strand transfer inhibitor; NNRTI, non-nucleoside reverse transcriptase inhibitor; NRTI, nucleoside reverse transcriptase inhibitor; PI, protease inhibitor.

| Mutation against drug class |  |
| --- | --- |
| NRTI | 129/321 (40.2%) |
| NNRTI | 150/321 (46.7%) |
| PI | 29/321(9.0%) |
| INSTI | 13/321 (4.0%) |
| Mutation classification |  |
| Major | 228/321 (71.0%) |
| Accessory | 93/321 (29.0%) |

Appendix table 5: Resistance-associated mutation positions of participants with two available GRTs (n=34). RAMs only observed at one time point are shown in bold.

| ID | **ART** at first GRT | **ART** at second GRT | **NRTI** RAMs at first GRT | **NRTI** RAMs at second GRT | **NNRTI** RAMs at first GRT | **NNRTI** RAMs at second GRT | **PI** RAMs at first GRT | **PI** RAMs at second GRT | **INSTI** RAMs at first GRT | **INSTI** RAMs at second GRT |
| --- | --- | --- | --- | --- | --- | --- | --- | --- | --- | --- |
| 1 | ABC/3TC/LPVr | ABC/3TC/LPVr | NA | NA | K101E, E138A, G190A | K101E,  E138A,  G190A | L33F | L33F | **Q95K** | NA |
| 2 | ABC/3TC/LPVr | ABC/3TC/LPVr | NA | NA | K103N | K103N | NA | NA | NA | NA |
| 3 | ABC/3TC/LPVr | ABC/3TC/DTG | M184V | M184V | NA | NA | NA | NA | NA | NA |
| 4 | ABC/3TC/DTG | ABC/3TC/DTG | NA | NA | NA | NA | NA | NA | NA | NA |
| 5 | ABC/3TC/DTG | ABC/3TC/DTG | NA | NA | NA | NA | NA | NA | NA | NA |
| 6 | ABC/3TC/LPVr | ABC/3TC/DTG | A62V,  M184V | A62V,  M184V | V106M,  V179D,  F227L | V106M,  V179D,  F227L | NA | NA | NA | NA |
| 7 | ABC/3TC/LPVr | AZT/3TC/LPVr | L74V,  Y115F,  M184V | **M41L,**  L74V,  Y115F,  M184V | K103N, P225H | K103N, P225H | NA | NA | NA | NA |
| 8 | ABC/3TC/LPVr | ABC/3TC/DTG | **M184V** | NA | K103S,  V106A,  P225H | **K103N, K103R,** K103S, V106A, P225H | NA | NA | NA | NA |
| 9 | ABC/3TC/LPVr | AZT/3TC/LPVr | M184V | M184V | V106I,  V106M,  G190A | V106I,  V106M,  G190A | NA | NA | NA | NA |
| 10 | ABC/3TC/LPVr | AZT/3TC/DTG | NA | NA | NA | NA | NA | NA | NA | NA |
| 11 | ABC/3TC/LPVr | ABC/3TC/LPVr | **M184V** | **T69D** | NA | NA | NA | NA | NA | NA |
| 12 | TDF/3TC/DTG | TDF/3TC/DTG | M41L,  **M184V** | M41L | A98G | A98G | NA | NA | NA | NA |
| 13 | ABC/3TC/DTG | ABC/3TC/DTG | NA | NA | K103N,  K103S,  **G190A,**  **P225H** | K103N,  K103S | NA | NA | NA | NA |
| 14 | TDF/3TC/DTG | TDF/3TC/DTG | NA | NA | **E138K** | NA | NA | NA | NA | NA |
| 15 | TDF/3TC/DTG | TDF/3TC/DTG | NA | NA | K103N, P225H | K103N,  P225H | NA | NA | NA | NA |
| 16 | ABC/3TC/LPVr | ABC/3TC/LPVr | NA | NA | NA | NA | NA | NA | NA | NA |
| 17 | ABC/3TC/LPVr | ABC/3TC/LPVr | NA | **M184V** | NA | **K103N** | NA | NA | NA | NA |
| 18 | ABC/3TC/LPVr | ABC/3TC/LPVr | **M41L,**  M184V | M184V | Y181C,  H221Y | Y181C,  H221Y | NA | NA | NA | NA |
| 19 | ABC/3TC/LPVr | ABC/3TC/LPVr | M184V | M184V | **K103S** | **K103N** | NA | NA | NA | NA |
| 20 | ABC/3TC/LPVr | AZT/3TC/LPVr | M184V | M184V | NA | NA | NA | NA | NA | NA |
| 21 | ABC/3TC/LPVr | AZT/3TC/LPVr | M184V | M184V | NA | NA | NA | NA | NA | NA |
| 22 | ABC/3TC/LPVr | ABC/3TC/DTG | NA | NA | A98G, Y181C | A98G, Y181C | NA | NA | NA | NA |
| 23 | AZT/3TC/LPVr | AZT/3TC/LPVr | M41L,  M184V,  **T215F** | M41L,  **D67N,**  **K70R,**  M184V | NA | NA | L10F,  L33F,  I54V,  Q58E,  L76V,  V82A | L10F,  L33F,  **M46I,**  I54V,  **Q58E,**  L76V,  V82A | NA | NA |
| 24 | ABC/3TC/LPVr | ABC/3TC/LPVr | NA | NA | NA | **G190A** | NA | NA | NA | NA |
| 25 | ABC/3TC/LPVr | ABC/3TC/LPVr | NA | **D67N** | NA | NA | NA | NA | NA | NA |
| 26 | AZT/3TC/LPVr | TDF/3TC/DTG | M184V | M184V | NA | NA | NA | NA | NA | NA |
| 27 | ABC/3TC/LPVr | ABC/3TC/DTG | **L74V,**  **M184V** | NA | NA | NA | NA | NA | E157Q | E157Q |
| 28 | ABC/3TC/LPVr | ABC/3TC/LPVr | D67G,  M184V,  K219E | D67G,  M184V,  K219E | A98G, E138A, Y181C | A98G, E138A, Y181C | **L10F,**  I54V,  V82A | I54V,  V82A | **E157Q,**  **G163K** | NA |
| 29 | ABC/3TC/DTG | ABC/3TC/DTG | T215A,  **T215I,**  T215V | T215A,  T215V | NA | NA | NA | NA | NA | NA |
| 30 | TDF/3TC/DTG | TDF/3TC/DTG | NA | NA | NA | NA | NA | NA | NA | NA |
| 31 | ABC/3TC/DTG | TDF/3TC/DTG | NA | NA | E138Q,  G190A | E138Q,  G190A | NA | NA | NA | **E138K** |
| 32 | ABC/3TC/ATVr | TDF/3TC/DTG | L210W | L210W | NA | NA | NA | NA | NA | NA |
| 33 | TDF/3TC/DTG | TDF/3TC/DTG | NA | NA | K103N | K103N | NA | NA | NA | NA |
| 34 | TDF/3TC/DTG | TDF/3TC/DTG | NA | NA | K103N | K103N | NA | NA | NA | NA |

ART, antiretroviral therapy; INSTI, integrase strand transfer inhibitor; NNRTI, non-nucleoside reverse transcriptase inhibitor; NRTI, nucleoside reverse transcriptase inhibitor; PI, protease inhibitor; RAM, resistance-associated mutation
